# Flame Retardancy of Short Flax Fibers Modified by Radiation-Induced Grafting of Phosphonated Monomers: Comparison between Pre- and Simultaneous Irradiation Grafting

**DOI:** 10.3390/molecules29051176

**Published:** 2024-03-06

**Authors:** Clément Brendlé, Roland El Hage, Jean-Louis Clément, Sophie Rouif, Rodolphe Sonnier, Belkacem Otazaghine

**Affiliations:** 1 PCH, IMT–Mines Alès, 6, Avenue de Clavières, 30100 Alès, France; clement.brendle@mines-ales.fr (C.B.); roland.el-hage@mines-ales.fr (R.E.H.); rodolphe.sonnier@mines-ales.fr (R.S.); 2 ICR, CNRS, Aix-Marseille University, 13007 Marseille, France; jean-louis.clement@univ-amu.fr; 3 Ionisos SAS, 13 Chemin du Pontet, 69380 Civrieux-d’Azergues, France; sophie.rouif@ionisos.com

**Keywords:** flax, natural fibers, flame retardancy, radiation-induced grafting, e-Beam, γ rays, pre-irradiation, simultaneous irradiation

## Abstract

Short flax fibers have been modified by radiation-induced grafting using methacrylate monomers containing phosphorus to give them a flame-retardant character. Two methodologies, namely pre-irradiation and simultaneous irradiation grafting, were examined. Certain parameters, notably the dose and the irradiation source (e-Beam and γ rays), were evaluated. The grafting efficiency, in terms of phosphorus content (mass percentage), was measured by X-ray fluorescence spectrometry (XRF). Using simultaneous irradiation, 2.39 wt% phosphorus could be obtained from 10 kGy, compared to 100 kGy in pre-irradiation. Furthermore, for similar phosphorus levels, the location of the grafted polymer chains was different for the two methodologies. The effect of phosphorus content on thermal properties and fire behavior was evaluated on a microscopic scale using a pyrolytic flow combustion calorimeter (PCFC) and on a laboratory scale using a cone calorimeter. It was then pointed out that flammability was linked to the phosphorus content and likely its location, which is associated with the radiation-induced grafting methodology, showing that the grafting conditions influence the final fire properties. Simultaneous irradiation, thus, proved to be more interesting in terms of efficiency and final properties.

## 1. Introduction

In a context where demand is gradually moving toward more environmentally friendly materials, the use of renewable resources is taking on a growing significance. Due to their economic and environmental performance, plant-based natural fibers [1] are of particular interest in many fields of application [2,3]. To an extent, they may even be good candidates for replacing synthetic fibers in many composite applications [4,5]. Among different existing feedstocks, flax (*Linum usistatissimum*) has been widely investigated. It is not only one of the earliest domesticated plants [6] but it also offers a great combination of availability, low cost, light weight, high strength, stiffness, and annual production yield. Flax stalks contain fibers that provide their structural integrity. These fibers are multilayer assemblies made up of cellulose microfibrils incorporated in a matrix composed of hemicellulose and pectins [7,8]. Usually, flax fibers are extracted in the form of bundles [9] and can be used to produce many materials from everyday clothing to technical composites [7].

As with most plant materials, flax fibers have significant drawbacks, including moisture sensitivity [10] and flammability, impairing the final material’s performance [11]. To meet the requirements of some applications, it is sometimes necessary to modify these fibers. Many physical and chemical techniques have been developed to modify plant fiber [12,13,14]. Among all these methods, graft copolymerization is an interesting technique increasingly used to modify a substrate permanently. This technique involves using functional monomers to obtain grafted polymer chains, producing a material with improved physico-chemical properties while retaining its intrinsic characteristics. To do so, several methodologies may be used [15]. Among them, radiation-induced grafting is an appealing method. It uses ionizing radiation (usually e-Beam or γ rays) to produce active species (radicals) within a substrate, which can trigger graft polymerization reactions. Two mechanisms, known as pre-irradiation and simultaneous irradiation grafting, are used. Pre-irradiation grafting involves two steps. The substrate is first irradiated, producing active sites on the substrate, which can later be used to initiate the polymerization of an appropriate monomer. When irradiation is conducted under air atmosphere (through the “peroxidation method” [16]), hydroperoxides are formed from the reaction between radicals and oxygen molecules. The grafting reaction is then expected to be initiated both by the radicals produced on the substrate and also (when the temperature is sufficient) by the thermal dissociation of the peroxide species. However, due to the short lifespan of radical species [17,18] cold storage (sub 0 °C) is necessary before the grafting reaction to maximize the number of active species still present when the fibers are brought into contact with monomer and, thus, the grafting yield [19].

On the other hand, simultaneous irradiation (also called mutual irradiation) consists of irradiating the monomer and substrate simultaneously [20,21]. Usually, this method is carried out in a solvent, preferably in the absence of oxygen. However, some researchers have proven that grafting is also possible by impregnating the substrate with a solution containing the monomeand removing the solvent before the substrate is exposed to ionizing radiation [22,23,24], which offers greater flexibility from an industrial point of view. Regardless of the method, many parameters concerning irradiation and grafting conditions are decisive in the success of grafting reactions, such as the following: dose [22,25,26], dose rate [21,27], atmosphere [28], monomer concentration [25,29,30], solvent [27], and temperature [31]. By tailoring these parameters, it is possible to adjust the grafting yields and, therefore, the final properties of the material. However, it should be highlighted that irradiation can led to chain scissions among polysaccharides of the fibers [32]. Therefore, it is necessary, for cost reasons and final properties [33], to limit the imparted dose.

This work uses radiation-induced grafting to impart flame retardancy to short flax fibers. A comparison was conducted between grafting by the pre-irradiation and mutual irradiation of a commercial phosphonic methacrylate substance (PolySurF^TM^HP). In addition, the effect of some operating conditions was investigated. In particular, the effects of the irradiation technology (e-Beam and γ rays) were evaluated for both methodologies. Finally, pyrolysis flow combustion calorimetry (PCFC) and cone calorimetry were performed to assess the efficiency of the treatments on the fire behavior of the treated samples at small and bench scales, respectively.

## 2. Results and Discussion

### 2.1. Effect of Irradiation on Flax Fibers

**Figure 1:**
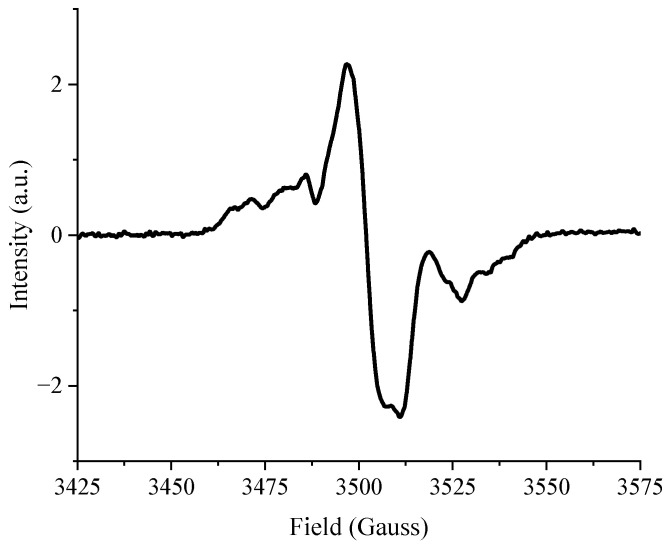
EPR spectra of SFF irradiated at 50 kGy.

Directly after irradiation, the short flax fibers (SFF) were stored in a refrigerator (−15 °C). Under these conditions, some radicals can persist for months after irradiation and can be detected by electron paramagnetic resonance (EPR) spectroscopy. The spectrum corresponding to e-Beam-irradiated SFF is shown in Figure 1. The overall appearance is consistent with previous work conducted on flax fabrics with similar operating parameters [26]. The side bands result from the contributions of C-centered radicals [18] and the main band arises from peroxyl radicals [34], formed by the reaction of C-centered radicals with oxygen.

**Figure 2:**
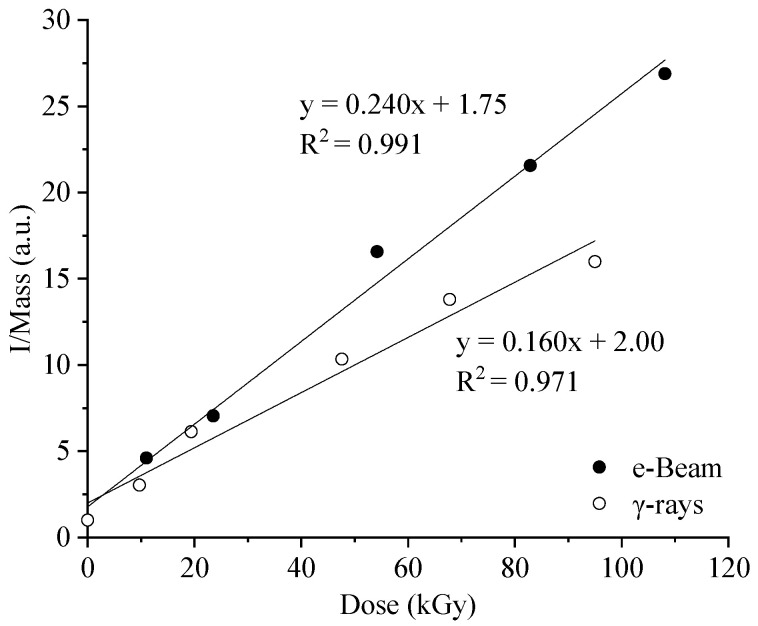
Evolution of the EPR signal intensities (double integration divided by the mass) with the imparted dose.

Figure 2 shows the evolution of the EPR signal intensity (I/Mass) as a function of dose. The I/Mass values give us an indication of the concentrations of the radicals formed within the fibers and permit us to establish a comparison between samples. The non-zero signal at 0 kGy corresponds to the radical species produced on flax fibers during the exposure of the plant to the sun or during the different post-harvest treatments. In our case, and independently of the radiation source, we observe a linear behavior over the dose range studied.

It should be noted that the slope for the trendline of the data series is lower in the case of γ irradiation than in the case of e-Beams. It is known that the concentration of radical species decreases rapidly at room temperature [18,35]. Because of a significantly lower dose rate than e-Beam, the residence time at room temperature in the radiation facility of fibers irradiated by γ rays is considerably higher, explaining a lower radical concentration. For example, while a few seconds are needed to reach 20 kGy with e-Beam, tens of hours are required with γ rays.

### 2.2. Pre-Irradiation Grafting

**Figure 3:**
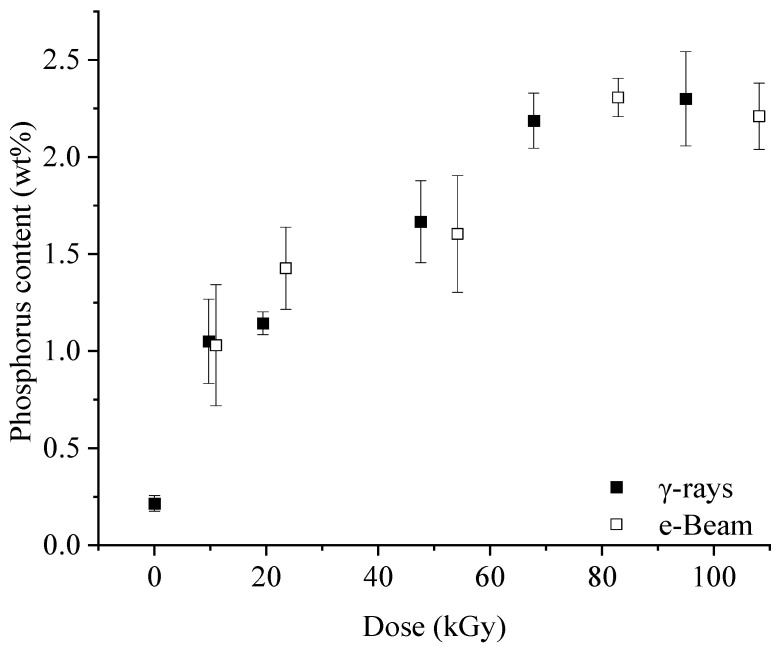
Phosphorus content as a function of dose depending on the radiation source.

Once treated, the fibers were washed thoroughly, and after drying, the phosphorus content was measured by X-ray fluorescence spectrometry. Note that even untreated SFF contains a small amount of phosphorus (about 0.07 wt%), which can be attributed to the phosphorus naturally present in the plant. Given that the concentration of active species is dose-dependent, the grafting rate is controlled by this parameter [26,36]. Indeed, the results presented in Figure 3 show that an increase in dose leads to a rise in the grafted amount, independent of the radiation technology. The phosphorus content goes from 1.05 ± 0.21 wt% at 10 kGy to 2.3 ± 0.24 wt% at 100 kGy with e-Beam and from 1.03 ± 0.31 wt% at 10 kGy to 2.21 ± 0.17 wt% at 100 kGy with γ rays. At about 80 kGy, a maximum is reached, possibly because of reduced monomer access to grafting sites due to their consumption in grafting and the competitive homopolymerization reaction [37]. This result shows that irradiation technology has no significant influence on grafting, even though a higher concentration of radicals has been measured with e-Beam for an equivalent dose. Graft polymerization is triggered both by radicals still present when the monomer is introduced and eventually by the dissociation of peroxides [19,34,38]. The dissociation of peroxides is not systematic, and additional work would be necessary to prove their contribution in our case. Concerning the radicals, it has been proven that the radicals involved in the initiation of grafting are “stable” and resistant to contact with water [39]. The differences in relative radical concentration shown in Figure 2 are partly caused by the quenching of the less stable radicals. Thus, it can be assumed that the quantities of radicals capable of initiating the reaction are approximately similar for the two technologies, explaining the results obtained following pre-irradiation.

A sample of unirradiated flax underwent the same treatment, showing that a small amount of phosphorus remained (0.21 ± 0.04 wt%). It is important to note that phosphoric acid molecules can be grafted onto cellulose [40,41]. Still, our conditions being much milder than those used by these authors, it is unlikely to occur. Then, this certainly arises from a small amount of monomer trapped in the structure of SFF.

**Figure 4:**
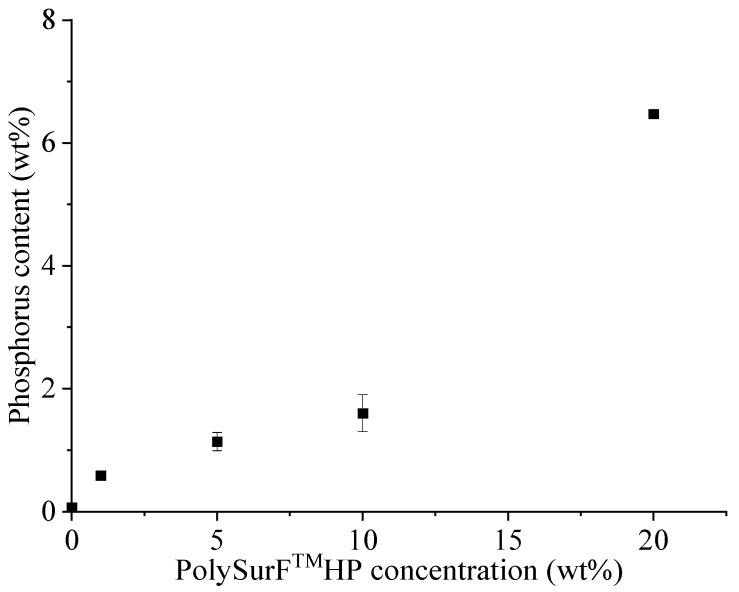
Effect of PolySurF^TM^HP concentration on final phosphorus content (wt%).

The influence of concentration on the grafted amount is shown in Figure 4. Note that PolySurF^TM^HP is not entirely soluble in water; we obtain a cloudy solution for concentrations above 10 wt% at room temperature. However, proper stirring allows a satisfying dispersion at ambient temperature and a limpid solution at 80 °C. As is often the case, monomer concentration positively influences grafting. Thus, higher phosphorus content was obtained by increasing the PolySurF^TM^HP concentration from 0.59 ± 0.04 wt% for 1 wt% concentration to 6.47 ± 0.07 wt% for 20 wt%. It should be noted that in the case of 20 wt% solution, a gel forms during the reaction. This may be caused by the cross-linking of PolySurF^TM^HP, due to the presence of bis(methacryloyloxyethyl) hydrogen phosphate in the methacrylate mixture, which can act as a crosslinking agent.

**Figure 5:**
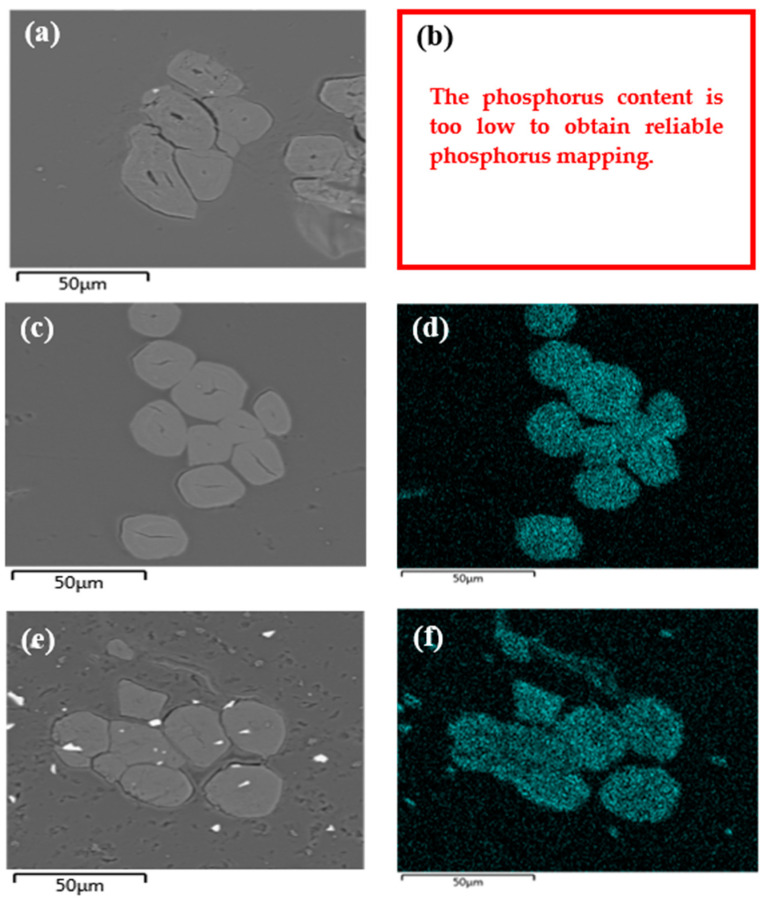
SEM observations and phosphorus imaging (EDX) of (**a**,**b**) untreated SFF; and pre-irradiation-treated SFF at 50 kGy by (**c**,**d**) e-Beam (P content = 1.88 wt%) and (**e**,**f**) γ rays (P content = 1.97 wt%).

Moreover, the peroxide dissociation leads to the release of hydroxyl radicals (ROOH → RO^•^ + ^•^OH), which may initiate undesirable polymerization reactions within the reaction medium. The high concentration of PolySurF^TM^HP may then promote the polymerization reactions triggered by the hydroxyl radicals. The formation of free polymer chains with possible cross-linking due to the presence of the di-methacrylate monomer leads to the formation of the observed gel. For practical reasons, it is preferable to remain at a concentration of 10 wt%. The distribution of phosphorus within the SFF cross-section was observed by SEM-EDX analyses (Figure 5). The maps show that flax fibers treated by pre-irradiation with PolySurF^TM^HP contain phosphorus homogeneously dispersed in the bulk of the fibers. This means that the monomers penetrated the structure and could be grafted into the bulk of the fibers.

The white spots present on the surface (Figure 5e) correspond to pollution resulting from the polishing steps.

### 2.3. Simultaneous-Irradiation Grafting

**Figure 6:**
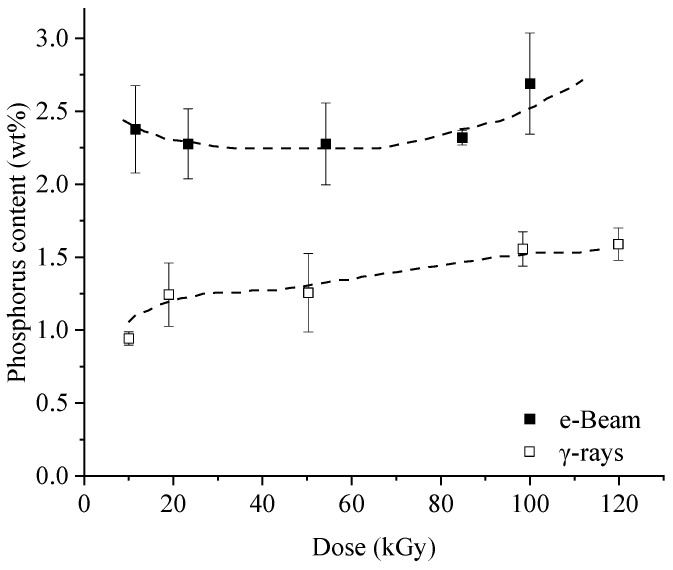
Phosphorus content as a function of dose and radiation source.

For simultaneous irradiation, the method used is close to that of previous work [22,23,41]. SFF were dipped for 5 min in a 10 wt% aqueous solution of PolySurF^TM^HP. After drying at room temperature and before irradiation, the average mass gain was 53.7 ± 4.2%. After irradiation, the flax fibers were thoroughly washed to remove the unreacted monomer units and the ungrafted polymer chains. The measurements of phosphorus content (Figure 6) show that contrary to pre-irradiation, irradiation technology influences the results obtained. Indeed, the P content obtained with e-Beam irradiation is systematically superior to that obtained by γ radiation, varying between 2.38 ± 0.30 wt% and 2.69 ± 0.35 wt% and between 0.97 ± 0.04 wt% and 1.59 ± 0.11 wt%, respectively. This result is assigned to the extent of oxygen inhibition reactions [27,28], which are favored by long residence times and low dose rates associated with γ radiation. It should also be noted that dose dependence is unclear regardless of the irradiation technology. Indeed, in e-Beam, 10 kGy allowed a graft of 2.39 ± 0.30 wt% while 100 kGy made it possible to obtain 2.69 ± 0.39 wt% of phosphorus, and the same tendency may be observed with γ rays. This trend has been observed in other studies [21,28] and was attributed to the occurrence of competitive phenomena (polymerization of self-initiated monomers). Since the irradiation step is conducted in the absence of solvents, this phenomenon can also be caused by the lack of molecular mobility, which, thus, limits the quantity of monomer in contact with the structure of the flax and the possibility of grafting. The effect of dose would perhaps be seen at lower doses, which may be interesting to study.

To evaluate the distribution of phosphorus within flax fibers, SEM-EDX analyses were conducted on samples treated by simultaneous e-Beam and γ irradiations. It should be noted that the distribution of phosphorus is different from that observed for the pre-irradiation strategy (Figure 5).

**Figure 7:**
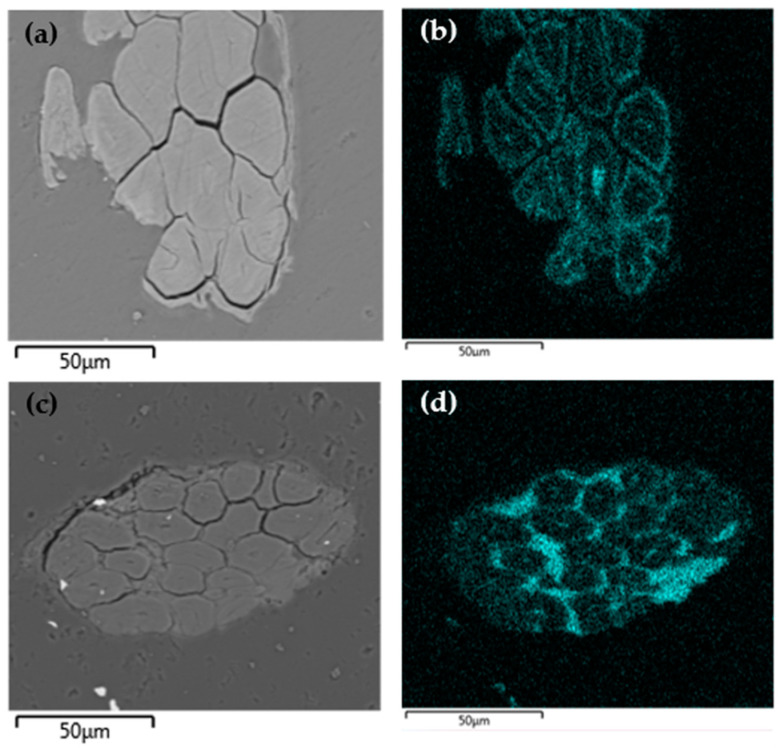
SEM observations and phosphorus imaging using EDX of SFF treated by simultaneous irradiation using (**a**,**b**) γ rays (P content = 1.54 wt%) and (**c**,**d**) e-Beam (P content = 1.97 wt%) at 50 kGy.

For samples treated by simultaneous irradiation with γ radiation (Figure 7b), phosphorus appears to be more concentrated in the outer part of the elementary fibers and their lumen. This effect is even more pronounced for samples treated by simultaneous irradiation with e-Beam (Figure 7d). The polymer appears to have filled the inter-fiber space and to stick the elementary fibers together. PolySurF^TM^HP is presented by the manufacturer as a solvent-free UV-curable additive. Therefore, its irradiation with ionizing radiation seems to cause its crosslinking and the fixation of monomers, which have not penetrated the structure of the fibers, explaining the observations we have just described.

### 2.4. Comparison between Pre- and Simultaneous Irradiation

The fibers adopted a pale tint after the pre-irradiation treatment, while fibers treated by simultaneous irradiation obtained a reddish color. Regarding final phosphorus content, it is evident that simultaneous irradiation using e-Beam leads to better results. Indeed, even though the SFF to PolySurF^TM^HP ratio is higher in the case of simultaneous irradiation (1:1 against 1:2 for pre-irradiation), the grafted amount is greater for doses between 10 and 50 kGy. We obtained 2.39 ± 0.30 wt% P at 10 kGy by simultaneous irradiation against 1.03 ± 0.31% using pre-irradiation. Considering the simplicity associated with simultaneous irradiation compared to pre-irradiation, we can conclude that simultaneous irradiation using e-Beam is preferable to obtain high phosphorus content. Using γ rays, however, higher phosphorus content was obtained by pre-irradiation (2.31 ± 0.10 wt% P at 75 kGy against 1.56 ± 0.12 wt% P at 100 kGy for simultaneous irradiation at 50 kGy). Still, experiments with the same SFF to PolySurF^TM^HP ratio need to be conducted to confirm this result.

### 2.5. Effect on the Flammability of SFF

**Figure 8:**
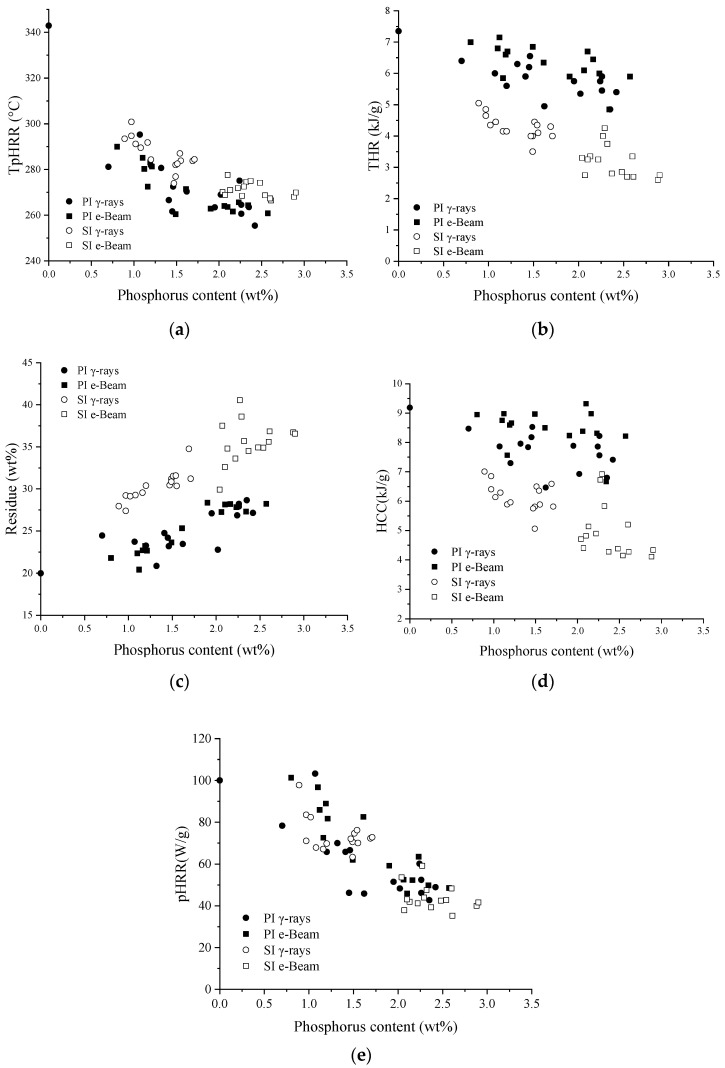
Main data from PCFC. (**a**) TpHHR, (**b**) THR, (**c**) Residue, (**d**) HCC, and (**e**) pHRR as functions of final phosphorus content (wt%). SI = simultaneous irradiation and PI = pre-irradiation.

A Pyrolysis Combustion Flow Calorimeter (PCFC) was used to compare the final properties of treated SFF regarding flame retardancy and thermal degradation at the micro scale. The results, in terms of total heat release (THR), peak heat release rate (pHRR), temperature at the peak (TpHRR), residue, and heat of complete combustion (HCC) as functions of the phosphorus content, are displayed in Figure 8.

**Figure 9:**
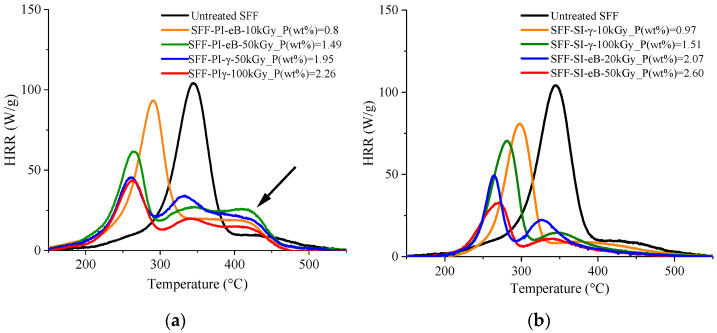
HRR curves for (**a**) pre-irradiation grafting and (**b**) simultaneous-irradiation grafting at various doses (eB = e-Beam).

For raw SFF, the results obtained were pHRR = 100 W/g, THR = 7.3 kJ/g, and TpHRR = 343 °C, which are comparable to the values obtained in other studies. Including ref. [42] (pHRR = 115 W/g, THR = 5.8 kJ/g, and temperature at pHRR = 371 °C), ref. [26] (pHRR = 230 W/g, THR = approx 9.0 kJ/g, and temperature at pHRR = 370 °C), and ref. [22] (pHRR = 160 W/g, THR = approx 8 kJ/g, and temperature at pHRR = 350 °C). These small variations may be attributed to the differences in composition between the substrates. Indeed, our materials contain a mixture of shives and fibers, which changes the overall chemical composition of our samples in addition to variations due to growing and harvesting conditions. Whether in pre-irradiation or simultaneous irradiation, the grafting of PolySurF^TM^HP leads to a decrease in THR and TpHRR while the char yield increases. This is because phosphorus promotes and catalyzes charring, leading to the thermal destabilization of the flax fibers and higher char yield [24]. Figure 8 shows that for each grafting strategy, all fire behavior parameters are controlled by the phosphorus content regardless of the grafting methodology, which is in accordance with previous works [22,30]. It is interesting to note that the results depend on the method used for a similar phosphorus content. Indeed, for similar phosphorus content, the results are always better with simultaneous irradiation than with pre-irradiation. The HCC and the THR are lower, while the char yield and the TpHRR are higher, meaning that samples treated in this way are better in terms of flame retardancy and thermal stability. For example, in pre-irradiation (e-Beam) we obtain HCC = 8.2 kJ/g, THR = 5.9 kJ/g, pHRR = 49 W/g, TpHRR = 261 °C, and char yield = 28.3% for a P content of about 2.6 wt%. On the other hand, in simultaneous methodology, we obtain HCC = 4.3 kJ/g, THR = 2.8 kJ/g, pHRR = 39 W/g, TpHRR = 275 °C, and char yield = 34.5% for a phosphorus content of about 2.4 wt%. The HRR curves’ overall shapes depend on the methodology (Figure 9). One can notice a supplementary shoulder at higher temperatures between 400 and 470 °C for pre-irradiated samples (indicated by the black arrow). This may be explained by the further decomposition of the residue at elevated temperature, i.e., an instability of the char formed. The decomposition also starts earlier in the case of pre-irradiation, meaning that the thermal stability of the sample treated by this method is lower than that of samples treated by simultaneous-irradiation methodology. These differences can be caused by variations in the distribution of phosphorus within samples; differences in the number average molar mass (Mn), and polydispersity (Ip) of the polymer formed; and operating conditions that can degrade the fibers.

**Figure 10:**
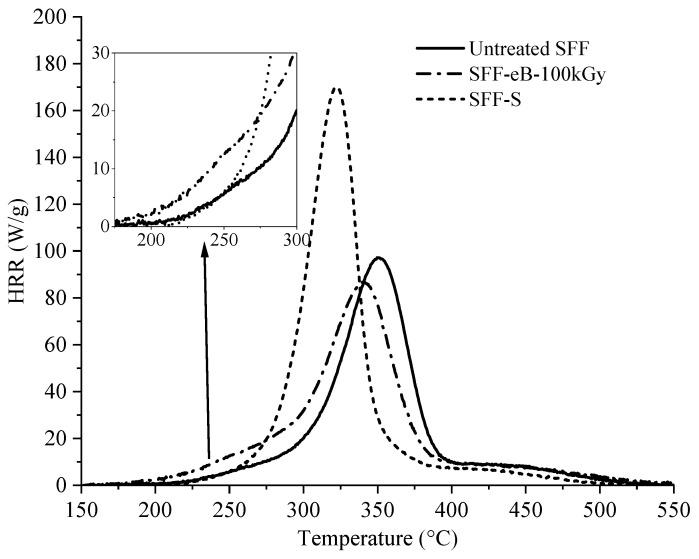
HHR curves for untreated SFF, 100 kGy-irradiated SFF, and PolySurF^TM^HP-soaked unirradiated SFF.

Additional experiments were carried out to separate the effect of irradiation and the solvent treatment’s impact on SFF. PCFC analysis was performed on 100 kGy e-Beam-irradiated fibers (SFF-eB-100 kGy) without treatment with PolySurFTMHP and on unirradiated flax fibers soaked in the aqueous solution of PolySurFTMHP (10 wt%, 80 °C, 30 min, and washed) (SFF-S). The results are shown in Figure 10, and the primary data are available in Table 1.

The irradiation of flax at 100 kGy causes a slight decrease in all parameters (pHRR = 86 W/g; THR = 6.8 kJ/g; TpHRR = 340 °C). We can also notice that degradation starts earlier in the case of the irradiated sample, just as we already observed for pre-irradiation-treated SFF (Figure 9a). This is explained by the decomposition of cellulose and other materials of flax fibers to form lower molecular weight compounds during irradiation [43]. It is known that one of the drawbacks of pre-irradiation grafting is that direct exposure to ionizing radiation causes the degradation of the substrate. Nevertheless, as we worked with PolySurF^TM^HP in low quantities and without solvent in the case of simultaneous irradiation, we would expect to see the same effect. However, this was not the case, probably due to the cross-linking of PolySurF^TM^HP, which reduces the release of low molecular weight compounds.

Acidic conditions may also degrade the substrate to a much greater extent; the pH of the PolySurF^TM^HP aqueous solution was measured at 0.9, which affected the thermal behavior of SFF. The PCFC experiments for the SFF-S sample underlined an essential increase in the pHRR, THR, and HCC compared to untreated fibers (from 100 to 172 W/g, from 7.3 to 8.7 kJ/g, and from 9.1 to 10.5 kJ/g, respectively), and a thermal destabilization of the fibers as the TpHRR went from 344 °C to 316 °C. Hydrolyzing the polysaccharides constituting plant fibers is possible using phosphoric acid [44]. This reaction is expected to occur preferentially in hemicelluloses because of their short chain length, low hydrogen bonding, and crystallinity [45].

Even though our conditions are milder than those usually used to hydrolyze natural fibers, it is clear that PolySurF^TM^HP degrades the SFF. Moreover, the irradiation of plant materials (e.g., wood) enhances the solubility of the plant cellulose in various media due to chain fragmentation. Also, it enhances the ability of this cellulose to undergo acid hydrolysis [46]. Therefore, the sum of the effects of irradiation and acid solution causes even more pronounced damage to flax, explaining the results observed here.

**Figure 11:**
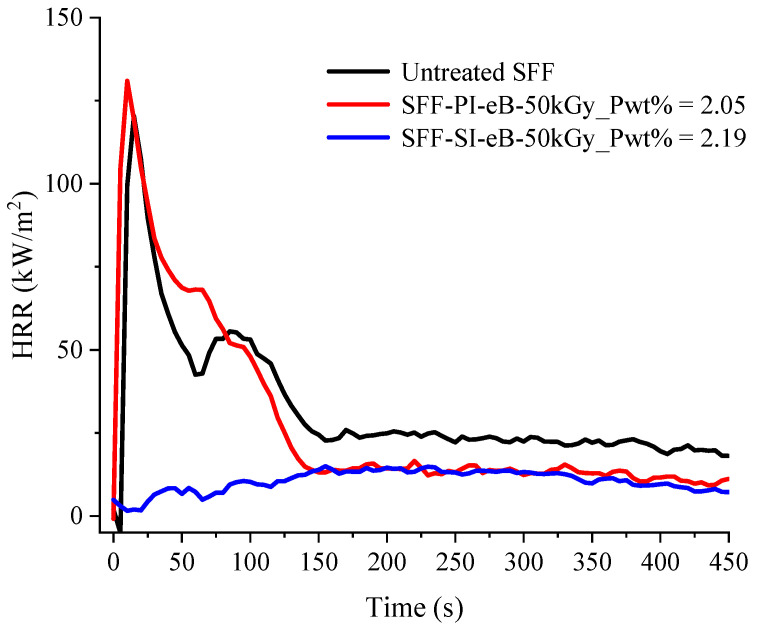
HRR curves for untreated SFF, pre-irradiation-treated SFF, and simultaneous irradiation-treated SFF using e-Beam at 35 kW/m^2^ irradiance.

The flame retardancy of untreated and PolySurF^TM^HP-treated SFF was also evaluated at a bench scale with a cone calorimeter. The HRR curves as a function of time at a heat flux of 35 kW/m^2^ of these samples are shown below (Figure 11). The data collected are presented in Table 2.

Measurements conducted on untreated SFF give TTI = 8 s, THR = 9.5 kJ/g, and pHRR = 120 kW/m^2^. In the case of the pre-irradiation-treated sample, the value of TTI decreases compared to untreated SFF (from 8 s to 2 s), and the residue increases (from 0.8 to 11.0%). This is in agreement with the findings of previous researchers [26,30].

For both samples, EHC is higher than the heat of complete combustion (HCC) measured using the PCFC. This is due to char thermo-oxidation, which occurs at the end of the test (after flame out) in the cone calorimeter but not in PCFC.

Despite the grafting of phosphorus, pHRR is not reduced for SFF-PI-eB-50 kGy. In previous work, we have shown that such insulating materials (as fibers in bulk) are thermally thin [47]. For such materials, pHRR is only dependent on initial mass, EHC, and heat flux. An equation was proposed to calculate its value roughly. In this equation, EHC is preferably estimated to be equal to the value measured in PCFC because thermo-oxidation (which modifies the value of EHC) is not effective when pHRR occurs a few seconds after ignition. Considering HCC values of 9.1 and 8.5 kJ/g for untreated flax and SFF-PI-eB-50 kGy, respectively, the calculated pHRR values are 107 and 100 kW/m^2^. These values are slightly lower but close to the measured pHRR.

**Figure 12:**
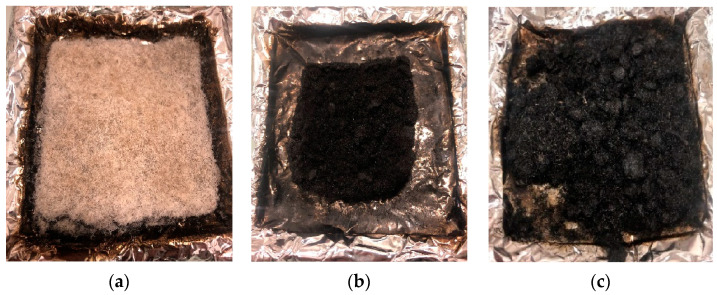
Residues after cone calorimeter tests of (**a**) untreated SFF, (**b**) pre-irradiation-treated SFF, and (**c**) simultaneous irradiation-treated SFF.

On the other hand, samples treated by simultaneous irradiation did not ignite during the cone test, even though thermal degradation (with a significant mass loss) effectively occurred. According to the criterion proposed by Lyon and Quintiere [48], ignition occurs when a “potential” heat release rate of 24 kW/m^2^ is reached. This critical HRR is the product of the mass loss rate and the heat of combustion. While the mass loss rate before ignition does not differ significantly for SFF-SI-eB-50 kGy compared to other samples (Figure S1, Supplementary Material), the difference is due to a much lower combustion heat, i.e., the nature of gases released. Indeed, according to Figure 8d, in PCFC, the HCC of this sample is close to 5 kJ/g (versus 8.5 kJ/g for SFF-PI-eB-50 kGy). This result is very surprising since previous studies stated that flammability is only controlled by the phosphorus content [30] and in the present case, the phosphorus content is similar for both samples. Consequently, the residue is much higher and reaches 23.5 wt% (P content = 2.19 wt%) using simultaneous irradiation. Phosphorus is known not only to promote charring but also to improve the thermo-oxidative stability of the char. This last effect can be seen both in the pre-irradiated and simultaneous-irradiated samples; indeed, even after flame out, when pyrolysis becomes aerobic, the char degradation rate is slow (Figure 12).

**Figure 13:**
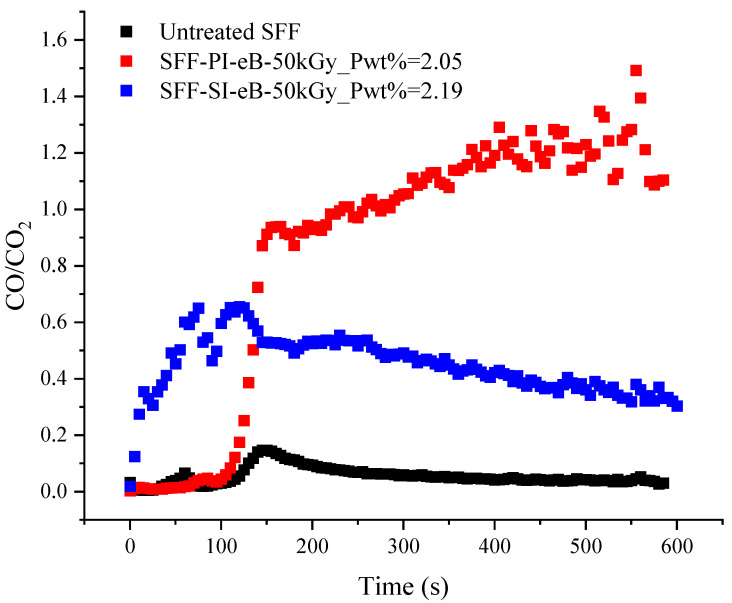
CO/CO_2_ as a function of time (measured by cone calorimetry).

Since cone calorimeter measurements are conducted in well-ventilated conditions, the EHC is usually close to the HCC measured by the PCFC. However, in our experiments, the EHC obtained by cone calorimetry is significantly higher than the HCC (12.4 kJ/g against 9.2 kJ/g for untreated SFF and 14.3 kJ/g against 7.9 kJ/g for PI-treated SFF (P = 2.24 wt%)). As already stated, this is due to the thermo-oxidation of the char after flame out. Usually, thermo-oxidation is accompanied by increased CO/CO_2_ ratios because it occurs at lower temperatures, and oxidation is incomplete. The CO/CO_2_ ratios measured during the cone calorimeter experiment are presented in Figure 13. It is noted that up to about 120 s, for untreated SFF, the CO/CO_2_ ratio is close to 0, meaning that the combustion is complete. After flame out, the CO/CO_2_ ratio increases momentarily before decreasing slowly until the residue is totally oxidized. In the case of a PI-treated sample, the combustion is also complete until around 110 s. After flame out, the CO/CO_2_ ratio increases sharply since the phosphorus improves char stability toward oxidation. Of course, for SFF-SI-eB-50 kGy, the CO/CO_2_ ratio is relatively high during the whole test. Indeed, only thermo-oxidation occurs because the sample does not ignite.

This difference in behavior for similar phosphorus content proves a difference in the final result for these treatments, both in terms of the location of the grafting, its structure, and the impact of the operating conditions on the substrate itself. These results show that for 50 kGy, simultaneous irradiation is the preferred method to impart flame retardancy to SFF by radiation-induced grafting using PolySurF^TM^HP.

## 3. Materials and Methods

### 3.1. Materials

Short flax fibers (SFF) of an average length of 2 mm were supplied by Addiplast (Saint-Pal-de-Mons, France). Their chemical composition, determined by chemical extractions (ASTM D 1102–84 for ash, ASTM D 1107–96 for extractives, ASTM D1104 for Holocellulose, ASTM D 1103–60 for cellulose, and ASTM D 1106–96 for Klason lignin), are presented below (Table 3). The high content of lignin is explained by the presence of shives mixed with the fibers.

**Figure 14:**
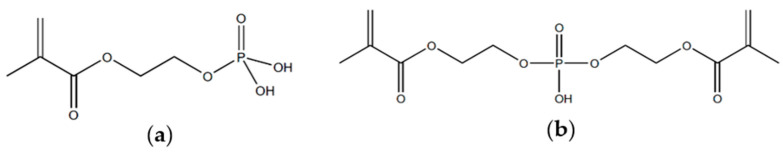
Monomers contained in PolySurF^TM^HP: (**a**) 2-(phosphonooxy) ethyl methacrylate and (**b**) bis(methacryloyloxyethyl) hydrogen phosphate.

PolySurF^TM^HP, a mixture of 2-(phosphonooxy)ethyl methacrylate (Figure 14a) and bis(methacryloyloxyethyl) hydrogen phosphate (Figure 14b) as well as 5 wt% of phosphoric acid, was received from Addapt Chemicals BV (Helmond, The Netherlands).

Nitric acid (HNO_3_, 70% purity) and sulfuric acid (H_2_SO_4_ to 98%) were purchased from CHEM-LAB (https://chemlabonline.com/) and used as received.

### 3.2. Grafting Procedure

#### 3.2.1. Irradiation of the Fibers

SFF were irradiated under air at room temperature. A first batch of fibers was irradiated using γ rays (^60^Co) at target doses of 10, 20, 50, 75, and 100 kGy by Ionisos SA (Dagneux, France) with an average dose rate of 2 kGy/h. Assigned doses were measured by alanine dosimetry with an uncertainty of 4.6%. Another batch of fibers was irradiated by e-Beam using anelectron accelerator (rhodotron) (energy 10 MeV, power 34 kW) at target doses of 10, 20, 50, 75, and 100 kGy, also by Ionisos SA (Chaumesnil, France). The dose rate was around 300 kGy/min. Thus, to avoid a rise in the temperature of the irradiated samples, the irradiation at doses greater than 20 kGy was divided into several passes at 25 kGy. Doses were measured by polystyrene calorimetry (uncertainty = 3.8%).

#### 3.2.2. Pre-Irradiation (PI) Grafting

Immediately after irradiation at the facility, the SFF was stored at −18 °C and then sent in refrigerated containers at the same temperature to preserve the radical species produced. Once received in the laboratory, the irradiated biomass was stored in a refrigerator (−15 °C) pending grafting manipulation. The target doses and the corresponding measured doses are displayed in Table S1. The grafting solution is a mixture of PolySurF^TM^HP and deionized water in adequate proportion according to the desired concentration. Before introducing the biomass, the solution was deoxygenated by bubbling with argon for 10 min. SFF (ratio fibers to solution 1:20 wt%) was introduced in the grafting solution, and bubbling was continued for two more minutes. The container was then tightly closed with a skirt cap, and the mixture was heated to 80 °C under stirring (300 revolutions/min) for 30 min. The treated fibers were washed two times with deionized water (30 mL, 80 °C) and twice with ethanol (30 mL, 78 °C) before being filtered onto filter paper. This step removes the unreacted monomer units and free polymer chains. The treated fibers were left to dry in a fume hood until their mass stabilized. Each manipulation was performed three times.

#### 3.2.3. Simultaneous Irradiation (SI) Grafting

A quantity of 1 g of SFF (fibers to solution ratio: 1:10) was mixed with a 10 wt% aqueous solution of PolySurF^TM^HP and soaked for 5 min with stirring (300 revolutions/min) at room temperature. The mixture was then filtered using cellulose paper and left to dry in a fume hood at room temperature until mass stabilization (around 24 h was necessary). Subsequently, a batch of impregnated SFF was irradiated under the abovementioned conditions. The target doses and the corresponding measured doses are displayed in Table S2. Finally, the irradiated flax fibers were washed two times with 30 mL of ethanol and two times with deionized water (80 °C, 300 revolutions/min) to remove unreacted monomer units, ungrafted oligomers, and polymer chains. Each manipulation was performed three times to ensure the reliability of the results.

### 3.3. Characterization

#### 3.3.1. Electron Paramagnetic Resonance (EPR) Spectroscopy

EPR analyses were made using a Bruker Elexsys E500 spectrometer, equipped with a Bruker SHQ EPR cavity. Glass NMR (Nuclear Magnetic Resonance) tubes (reference B-100-5-7 from Innova chem, Angervilliers, France) were filled with a known amount of irradiated material (about 100 mg) and then inserted into the EPR cavity before recording the spectra. The settings of the spectrometer were as follows: sweep time: 21 s; sweep width: 500 G, power: 10 mW, modulation amplitude: 1.0 G, conversion time: 20.52 ms, modulation frequency: 100 kHz. Signal intensities were measured by double integration of the signal divided by the mass of the sample (I/Mass).

#### 3.3.2. X-ray Fluorescence Spectrometry

The phosphorus content in treated flax fibers was determined using an Oxford XMET 5100 X-ray fluorescence spectrometer (Oxford Instruments, Abingdon, UK). The flax samples were first compressed into discs (1 mm thickness, 2.5 cm diameter) using a Prontopress-2 (Struers, Copenhaguen, Denmark) (30 kN, 110 °C for 3 min, then cooled for 2 min). The measurements were conducted under a normal atmosphere with the following settings: 13 kV, 45 μA (optimal for the kα band of phosphorus), and the analysis time was set at 1 min. The peaks’ intensities were converted into a mass fraction of phosphorus using a pre-established calibration curve (Figure S2). Each value in this paper corresponds to the average value of three measurements. 

#### 3.3.3. Scanning Electron Microscopy (SEM) Coupled with an Energy Dispersive X-ray Spectrometer (EDX)

Flax fibers were trapped in an epoxy resin (Bis-[4-(2,3-epoxypropoxy)phenyl]propane) that was crosslinked using a catalyst (100CC CATALYST REF IP) provided by Presi (Eybens, France) The material was then polished according to a well-defined protocol using a Mecatech 250 SPI automatic polisher of the same brand. The polished sections were then analyzed using a FEI Quanta 200 scanning electron microscope (FEI Company, Hillsboro, USA)to observe the sections of the fibers. The samples were observed under vacuum at a distance of 10 mm with a voltage of 12.5 kV. In order to locate phosphorus within the fibers section, energy-dispersive X-ray spectroscopy (EDX) mapping was generated using an Oxford INCA Energy system coupled to the SEM.

#### 3.3.4. Pyrolysis Combustion Flow Calorimetry (PCFC)

A combustion microcalorimeter (PCFC: Pyrolysis Combustion Flow Calorimetry) from Fire Testing Technologies (East Grinstead, UK) was used to evaluate the small-scale fire behavior of functionalized SFF. The samples (2–5 mg) underwent an anaerobic pyrolysis step (1° C·s^−1^) between 100 and 750 °C under a nitrogen flow rate of 100 mL min^−1^. After this step, the gases from pyrolysis were oxidized in the presence of a nitrogen–oxygen mixture (80:20), and the corresponding heat release rates were calculated according to the Huggett relation [49] (1 g of oxygen consumed = 13.1 kJ of energy released). The data collected are the peak heat release rate (pHRR), the temperature of this peak (TpHRR), and the total amount of energy released (total heat release = THR). After the experiment, the mass of the residue was measured to assess the amount of char formed, and finally, the heat of complete combustion (HCC) was calculated. The measurements were repeated twice to ensure the reliability of the results.

#### 3.3.5. Cone Calorimetry

A cone calorimeter (Fire Testing Technology) was used to assess the flammability at bench scale. The method used is inspired by a recent work [47]. Around 11 g of flax short fibers (untreated and treated) were placed in a 10 × 10 cm^2^ sample holder (the thickness of the batch was around 4 mm) and exposed to a 35 kW/m^2^ radiant heat flux in ventilated conditions (air flow = 24 L s^−1^). The distance between the cone heater and the sample surface was 2.5 cm, and no grid was used. During its thermal decomposition, the sample releases volatile gases, which are ignited using a spark igniter. In the same manner, as with PCFC, HRR was determined according to oxygen depletion [49]. Time-to-ignition (TTI), peak of heat release rate (pHRR), total heat release (THR), residue yield, and effective heat of combustion (EHC) were recorded.

## 4. Conclusions

This work has demonstrated the possibility of functionalizing short flax fibers with phosphonated polymer chains using a commercial methacrylates mixture (PolySurF^TM^HP) by radiation-induced grafting. Two methodologies were used: pre-irradiation and simultaneous irradiation grafting. The following conclusions could be drawn from this work:

- The type of irradiation (γ rays or e-Beam) does not influence the grafted amount in pre-irradiation but strongly influences the results obtained by simultaneous irradiation. In the latter case, using e-Beam led to a higher phosphorus content.
- Regarding final phosphorus content, simultaneous irradiation grafting is more effective than pre-irradiation grafting, especially at low doses. While phosphorus content reached around 1.03 ± 0.31 wt% at a low dose of 10 kGy in pre-irradiation, 2.38 ± 0.30 wt% was obtained at the same dose using simultaneous irradiation.
- SEM-EDX mapping proved that the location of the grafted molecules differs from the grafting methodology. In pre-irradiation, the phosphorus was homogeneously dispersed in the cross-section of the elementary flax fibers. In contrast, phosphorus was mainly located at the surface and around the fibers in simultaneous irradiation.
- Unlike previous work, flammability was not only controlled by the phosphorus content but also by the operating conditions. For 50 kGy and at equivalent phosphorus content, simultaneous irradiation led to better results than pre-irradiation on both the micro scale and the bench scale. Two complementary explanations can be proposed. First, the location of phosphorus (which depends on the grafting methodology) plays a role in flammability. Second, the irradiation of plant fibers followed by exposure to an acidic solution during the pre-irradiation grafting reaction causes the substrate’s deterioration, affecting its final properties.

## Figures and Tables

**Table 1:** Main results obtained by PCFC.

Sample	THR (kJ/g)	pHRR (W/g)	TpHRR (°C)	Residue (%)	HCC (kJ/g)
Untreated SFF (P(wt%) = 0.07)	7.3	100	344	20.1	9.1
SFF-eB-100 kGy (P(wt%) = 0.07)	6.7	87	343	19.3	8.3
SFF-S (P(wt%) = 0.17)	8.7	172	316	17.1	10.5

**Table 2:** Main data from cone calorimeter tests for untreated and PolySurF^TM^HP-treated SFF.

Sample	TTI (s)	pHRR (W/g)	THR * (kJ/g)	Residue (wt%)	EHC * (kJ/g)
Untreated SFF (P(wt%) = 0.07)	8	120	9.5	0.8	12.4
SFF-PI-eB-50 kGy (P(wt%) = 2.05)	2	131	10.4	11.0	14.3
SFF-SI-eB-50 kGy (P(wt%) = 2.19)	/	/	/	23.5	/

* THR, EHC and residue were measured 200 s after the beginning of the test and after the flameout.

**Table 3:** Chemical composition of flax short fibers under study.

Extractables (%)	Lignin (%)	Cellulose (%)	Hemicelluloses (%)	Ash Content (%)
2.9 ± 0.5	9.1 ± 0.6	76.2 ± 0.1	9.4 ± 0.1	2.2 ± 0.4

## Data Availability

Data are contained within the article and Supplementary Materials.

## Supplementary Materials

The following supporting information can be downloaded at https://www.mdpi.com/article/10.3390/molecules29051176/s1. Figure S1: Sample mass as a function of time during cone calorimetry testing.Figure S2: Calibration curve used to measure P content of SFF treated with XRF; Table S1: Target dose and corresponding measured dose for pre-irradiation; Table S2: Target dose and corresponding measured dose for simultaneous irradiation.
