# Supplementary material for: Flame Retardancy of Short Flax Fibers Modified by Radiation-Induced Grafting of Phosphonated Monomers: Comparison between Pre- and Simultaneous Irradiation Grafting
Source: Molecules. 2024 Mar 6;29(5):1176. doi: 10.3390/molecules29051176 (PMC10934551; doi:10.3390/molecules29051176)
Supplement: Supplementary file 1 [file molecules-29-01176-s001.zip › molecules-2895338-supplementary.pdf]

Supporting information

Table S1. Target dose and corresponding measured dose for pre-irradiation.

Target dose (kGy)	10	20	50	75	100
Measured dose (e-Beam) (kGy)	9.7	19.4	47.6	67.8	95.0
Measured dose (γ-rays) (kGy)	11	23.5	54.2	82.9	108.1

Table S2. Target dose and corresponding measured dose for simultaneous irradiation.

Target dose (kGy)	10	20	50	75	100
Measured dose (e-Beam) (kGy)	11.5	23.3	54.2	84.8	100
Measured dose (γ-rays) (kGy)	10	19	50.3	98.4	119.8

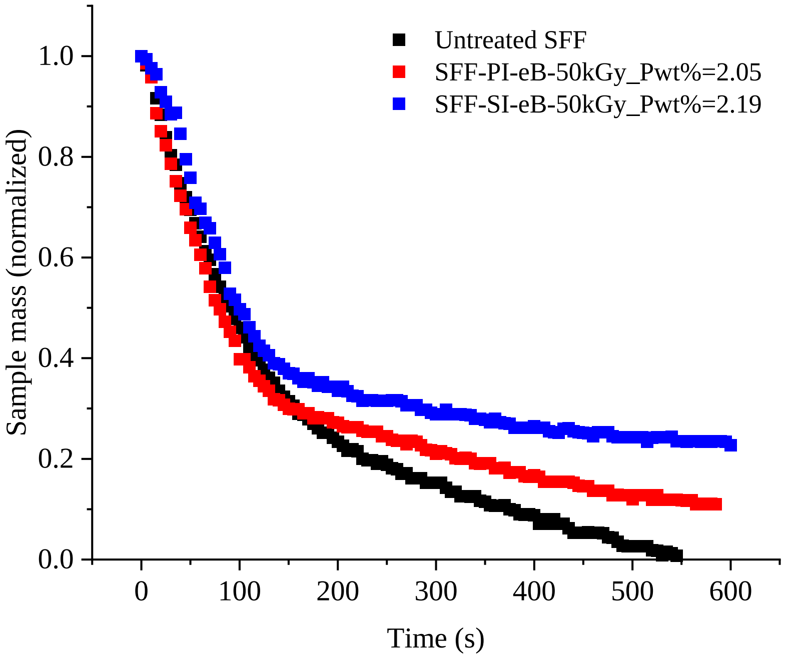

Figure S1. Sample mass versus time during cone calormetry testing.

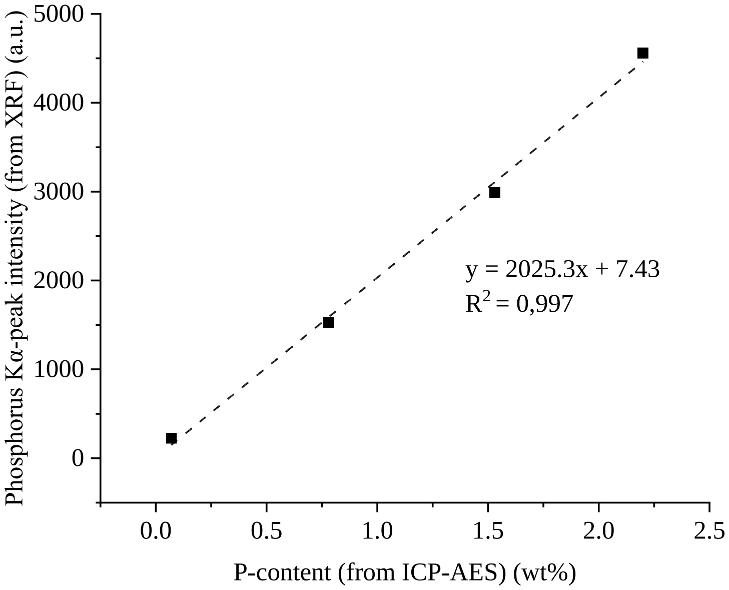

Figure S2. Calibration curve used to measure P-content of treated SFF with XRF.
